# Examining Older Adults’ Home Functioning Using the American Housing Survey

**DOI:** 10.3390/ijerph19084691

**Published:** 2022-04-13

**Authors:** Mi Jung Lee, Daejin Kim, Sergio Romero, Ickpyo Hong, Nikolay Bliznyuk, Craig Velozo

**Affiliations:** 1 Department of Nutrition, Metabolism & Rehabilitation Sciences, University of Texas Medical Branch at Galveston, Galveston, TX 77555, USA; 2 Department of Interior Design, Iowa State University, Ames, IA 50011, USA; daejink@iastate.edu; 3 Department of Occupational Therapy, University of Florida, Gainesville, FL 32610, USA; sromero@phhp.ufl.edu; 4 Veterans Rural Health Resource Center–Gainesville, Office of Rural Health, Gainesville, FL 32601, USA; 5 Department of Occupational Therapy, Yonsei University, Wonju 26493, Korea; 6 Department of Agricultural and Biological Engineering, University of Florida, Gainesville, FL 32611, USA; nbliznyuk@ufl.edu; 7 Division of Occupational Therapy, Medical University of South Carolina, Charleston, SC 29425, USA; velozo@musc.edu

**Keywords:** aging in place, American housing survey, functional independence, home safety, older adults

## Abstract

Identifying individuals at risk of experiencing functional difficulty at home would support timely home safety assessment and modification services, which could lead to reducing home incidents such as falls. The objective of this study was to calculate older adults’ functional difficulty at home scores using the 12 physical function items in the American Housing Survey National and Metropolitan Data (AHS). Among the 28,474 older adults selected for this study, we used 19,932 for measurement model development and 8542 for model testing. Confirmatory factor analysis confirmed an adequate fit of the one-dimensional model with all AHS 12 items loading on one latent construct (functional difficulty at home) (RMSEA: 0.034, CFI: 0.990, and TLI: 0.988). Based on our model selection process, we determined that the Graded Response Model was an optimal model for our analysis and separated two detected differential functioning items for each sex. Using the testing dataset, we validated that the estimated functional difficulty scores showed an expected item hierarchy and statistically significant differences in their association with housing and demographic conditions (*p* < 0.001). Our results demonstrated the process of using the 12 AHS physical function at home items to produce validated scores of older adults’ functional difficulty at home.

## 1. Introduction

Individuals can foster autonomy and independence when the home provides a safe and secure environment that supports a connection to their family members and to their communities [1]. Older adults overwhelmingly prefer to remain in their homes over living elsewhere, such as in assisted living facilities or in their children’s homes [2]. However, age-related diseases, frailty, and disability might prevent older adults from residing in the home safely since, in the home, they might experience falls or other accidents [3,4,5,6,7].

Home assessments conducted to determine the safety and suitability of a home for an older adult resident are thus generally performed by clinicians (e.g., occupational therapists) [3,8]. A home assessment is a comprehensive inspection of the home, its surrounding environment, and the lifestyle of the resident [9,10,11]. A home safety assessment is recommended for an individual who has had a recent decline in health but has safe functional performance in the home or whose primary caregiver is experiencing an increased caregiving burden [3]. During home assessments, the organic interactions between the person and the residential environment are examined to determine the necessary home modifications that can maximize the person–environment fit and help individuals maintain independence and quality of life [12,13].

Identifying and modifying potential home hazards (slippery floors, loosened carpets, clutter, and others) can substantially reduce the risk of falls and home accidents [14]. A Cochrane review by Gillespie et al. reported that professionally prescribed home hazard evaluations and modifications decrease the rate of falls by older adults who have a history of falling [14]. Similarly, Nikolaus and Bach concluded that a comprehensive geriatric assessment coupled with a home safety evaluation and the prescribed use of technical and mobility aids prevented 31% of falls (37% for subjects with a history of two or more falls in the previous year) as compared to a comprehensive geriatric assessment followed by usual home care (*p* < 0.05) [15]. Moreover, study participants who adopted one or more recommended home modifications experienced a significantly decreased rate of falls (Incident Rate Ratio = 0.64, CI = 0.37–0.99), whereas participants without any home modifications did not have a reduction in their rate of falls (Incident Rate Ratio = 1.05, CI = 0.82–1.41) [15].

However, a home assessment is a costly and labor-intensive practice that requires a home visit by a clinical expert to examine functional hazards and risks [8,16]. Providing timely home assessment and corresponding modification services might not be feasible for areas where clinicians are not available (e.g., rural areas) [3,17,18]. Innovative alternatives are needed to allow clinicians to efficiently and effectively assess the home environment in order to identify necessary modifications.

Additionally, individuals at risk of having functional difficulty at home have a higher likelihood of experiencing home accidents, which can lead to such unwanted consequences as injuries, hospitalization, or even death [19,20,21]. Thus, identifying individuals at risk of experiencing functional difficulty at home would result in targeted interventions that should improve outcomes. Simultaneously examining both housing features (e.g., steps, lighting, grab bars, and entry-level bathroom) and household member characteristics (e.g., household income, education, age, disabilities, monthly mortgage, and other demographic information) could yield a preliminary prediction of the probability of an individual having functional difficulty at home. Pre-screening individuals at risk of experiencing functional difficulty at home could encourage the use of timely home assessment and modification services, which, in turn, could reduce the incidence of home falls and ultimately reduce hospital admission rates.

This study is the first of three sequential studies which investigate the feasibility of using comprehensive nationwide housing and demographic data to develop a prediction model in order to identify elderly individuals at risk of experiencing functional difficulty at home. One primary step in this process to develop a prediction model is to estimate a client’s home functioning precisely. Thus, the purpose of this study was to calculate older adults’ functional difficulty at home using an optimal measurement model for the 12 physical function items in the American Housing Survey National and Metropolitan Data (AHS). The specific steps were as follows: (1) determine the item–factor structure of the 12 AHS physical function items, (2) select an optimal psychometric model for the 12 AHS physical function items and estimate the functional difficulty at home scores for each individual, and (3) validate the functional difficulty scores with external variables that are expected to have significant associations.

## 2. Materials and Methods

### 2.1. The American Housing Survey National and Metropolitan Data

The AHS is the largest biennially (e.g., 2011, 2013, and 2015) administered national survey that describes people and their homes, sponsored by the U.S. Department of Housing and Urban Development and conducted by the U.S. Census Bureau [22]. The AHS’s interviewers collect the national samples (approximately 116,000 samples) via calls or home visits. Participating housing units were scientifically selected and interviewed every two years to describe all housing units in the U.S. For this retrospective secondary data analysis study, we selected the 2011 AHS because health-related functional difficulty items were exclusively included in the 2011 survey.

### 2.2. Data Sample

For our sample, we selected older adults who were 65 years or older and who responded to the AHS questionnaires themselves (i.e., not by proxy) and extracted one older adult per household. The “CONTROL” variable represents each housing unit, with each household member assigned to the “PLINE” variable. A number of housing units include multiple “PLINE” variables. A new index variable was created by combining the “CONTROL” and “PLINE” variables to give each individual a unique code. We linked the “person” data table to the “newhouse” data table by using the “CONTROL” variable; then, we selected our target individuals using our new index variable. The “person” data table includes individual person-level information such as age, gender, education, income, and physical function at home. The “newhouse” dataset covers house-level information (e.g., handrails or grab bars in a unit, extra-wide doors/hallways, wheelchair accessible kitchen cabinets, unit-built year, and loose/broken/missing steps in common stairs).

### 2.3. Physical Function at Home Items

We retrieved 12 dichotomous physical function at home items to represent the level of functional difficulty at home (Table 1). These items are combinations of gross and fine motor functional movements at home with a dichotomous response option: (1) have difficulties or (2) have no difficulties.

### 2.4. Statistical Analysis

**Figure 1:**
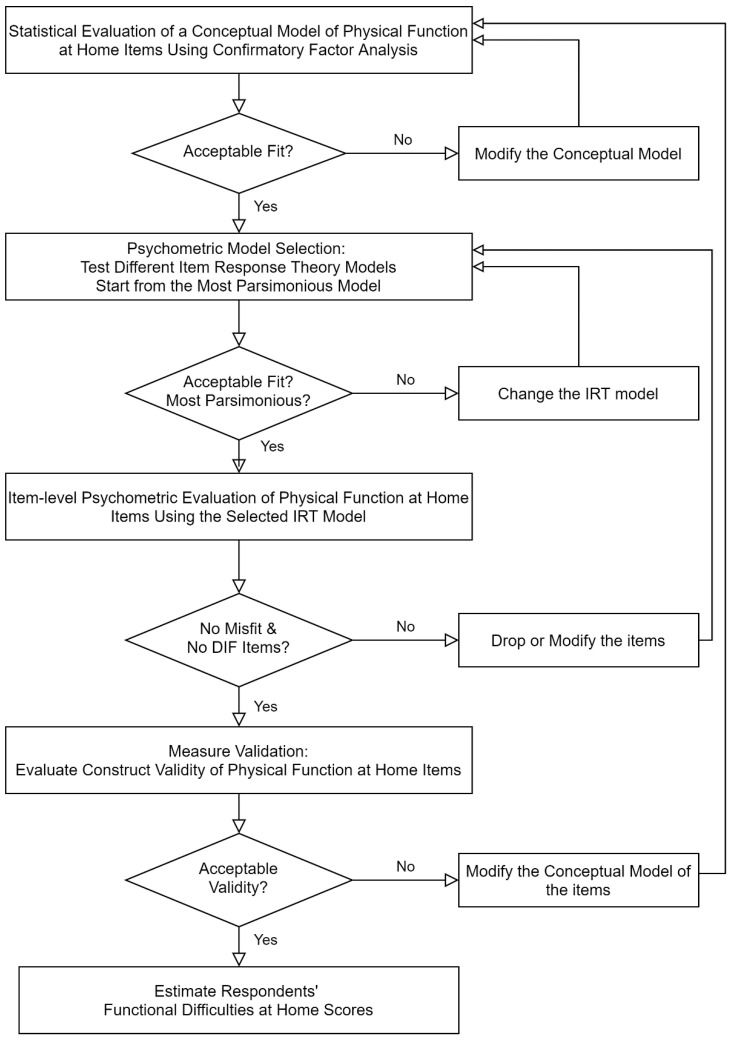
Model Selection and Validation Process.

The statistical analysis was threefold: (1) confirmation of the theoretical item–factor structure of the 12 AHS physical function at home items using confirmatory factor analyses (CFA) [23]; (2) estimation of older adults’ functional difficulty at home by applying the optimal psychometric model for the items using item response theory (IRT) analyses; and (3) validation of older adults’ functional difficulty at home scores by testing the construct validity with 7 demographic and 21 housing feature characteristics. We divided our sample into two groups (70% training and 30% testing). The identified theoretical item–factor structure and the optimal psychometric model based on the training dataset were validated with the testing dataset. The detailed statistical analysis process is described in Figure 1. For all statistical and analytic processes, R version 4.0.3 and R studio version 1.3.1093 were used with R packages (lavaan, summarytools, mirt, mirtCAT dplyr, tidyr, mgsub, foreach, and doParallel) [24,25,26,27,28,29,30,31,32,33].

#### 2.4.1. Confirmatory Factor Analysis

Using our training dataset, we conducted CFA to evaluate a theoretical structural model of the 12 physical function at home items, in which the variance in all 12 items is explained by one latent construct, “functional difficulty at home”. The following model fit criteria were used: (1) root mean square error of approximation (RMSEA < 0.08), (2) comparative fit index (CFI > 0.95), and (3) Tucker–Lewis Index (TLI > 0.95) [34]. We used weighted least squares mean and variance (WLSMV) to adjust the binary or ordered responses of the 12 physical function at home items and then performed listwise deletion for missing responses. Item factor loadings, coefficients of determination (R-squared), and local independence with a cutoff at 0.2 of residual correlations were also investigated [35].

#### 2.4.2. Measurement Model Selection

Several IRT models were tested. We began by fitting the simplest model to the AHS physical function at home items, then moved to increasingly more complex models until the model satisfied the adequate model fit indices (the same as the fit criteria used for confirmatory factor analysis). When multiple models demonstrated adequate fit indices, we conducted a Wald test to determine a statistically dominant model for the 12 AHS physical function at home items. Moreover, we evaluated differential item functioning (DIF) and item-fit statistics in order to produce optimal estimations of older adults’ functional difficulty at home.

DIF was tested across sex, age groups, income groups, and tenure type. The Wald test was used with the level of significance (*p*-value) at 0.05 and the Bonferroni correction to control for inflated Type I errors in multiple DIF tests. In addition, we controlled for group differences in functional difficulty at home in order to detect the true DIF items. For example, we expect that the 85-years-or-older group will have more severe functional difficulty at home than the two younger groups ((1) 65–74 years and (2) 75–84 years). We controlled for this group difference in functional difficulty at home in order to detect the true DIF items. Items confirmed to have DIF were divided according to the relevant category of DIF items to avoid bias in estimating functional difficulty at home [36]. For instance, if Item 1 is identified to have DIF between males and females, we would divide Item 1 into two items: Item 1 for males and Item 1 for females. These two items would then be used independently to estimate functional difficulty at home.

To detect misfit items, we used S-X2 for significant misfit items and RMSEA S-X2 for the magnitude of misfit items to our measurement model. Items with *p* < 0.001 of S-X2 and RMSEA S-X2 > 0.08 were classified as misfit items [37]. Empirical plots were also examined before the deletion of a misfit item. Finally, when a measurement model was finalized, the factor scores for each older adult were estimated using the confirmed measurement model of the 12 AHS physical function at home items.

#### 2.4.3. Measurement Validation

The construct validity of the AHS physical function at home items was evaluated by examining item contents in an item hierarchy and testing statistically significant differences in theoretically associated variables using our testing dataset. We hypothesized that the items related to gross motor movement were more challenging to perform than those related to fine motor movement for older adults with functional difficulty at home. For example, older adults with minimal to moderate functional abilities are expected to have difficulty in performing bending or stooping (gross motor), but not in using faucets or sinks (fine motor). However, older adults with severe functional difficulty at home would experience challenges in bending or stooping as well as in using faucets. In addition, the statistical differences in functional difficulty at home across different categories of all 28 demographic and general housing feature variables were tested. Of note, the sex differences of the functional difficulty at home were tested, controlling for age. In order to estimate older adults’ factor scores, each response of the 12 functional difficulty items was recoded from (1) have difficulties or (2) have no difficulties to (1) have no difficulties or (2) have difficulties, for the intuitive interpretation of functional difficulty level. Higher factor scores indicated more severe functional difficulty at home.

For this process, Kruskal–Wallis tests and Bonferroni corrections were conducted to adjust for the non-normal factor score distribution and inflated *p*-values from multiple comparisons.

## 3. Results

### 3.1. Demographics

Among a total number of 43,650 older adults, 28,474 older adults were selected for this study. The mean age of our sample was 75.1 ± 7.6 years. Females were the predominant sex in the sample (17,316, 60.8%). More than 45% (*n* = 12,911) of our sample had an annual family income of less than USD 25,000, and about 74% (*n* = 21,022) of our sample were homeowners. Of the selected older adults, 21.1% (*n* = 6018) had difficulty walking or climbing stairs (walking disability), 4.5% (*n* = 1272) had dressing or bathing difficulty (self-care disability), 4% (*n* = 1150) had both walking and self-care disabilities, and 76.4% (*n* = 21,767) reported no difficulties in either disability type. Moreover, our sample reported the following general health status: excellent (*n* = 5712, 20.1%), very good (*n* = 13,109, 46.0%), fair (*n* = 6987, 24.5%), and poor (*n* = 2070, 7.3%). Detailed information regarding the sample across age, sex, income, and tenure type, along with functional conditions, is provided in Table 2.

### 3.2. Confirmatory Factor Analysis

A total of 19,932 older adults in the training dataset were used for CFA. CFA confirmed that the one-dimensional model is adequate for the 12 AHS physical function at home items. The responding fit indices of the one-dimensional model for the 12 AHS physical function at home items were RMSEA (0.034), CFI (0.990), and TLI (0.988). All items were highly loaded on one latent construct (functional difficulty at home) (λ > 0.741), and the model explained more than 71% of the variance in all items except for Item 5 (Has difficulty using fingers to grasp small objects, 55%). All items supported the local independence assumption of the one-dimensional model (residual correlations, r < 0.20). A total of 19,440 older adults were used to test a theoretical structural model of the 12 physical function at home items after listwise deletion for missing responses (2.5%).

### 3.3. Measurement Model Selection

The 12 AHS physical function at home items did not demonstrate an adequate fit to the Rasch model (simplest model): RMSEA (0.928), TLI (−4.425), and CFI (0). We then investigated the fit of the 12 AHS items to two 2PL IRT models: the Generalized Partial Credit Model (GPCM) and the Graded Response Model (GRM). Fit indices for both GPCM and GRM were appropriate, with RMSEA (0.036 and 0.036), CFI (0.997 and 0.993), and TLI (0.996 and 0.992), sequentially. A subsequent Wald test indicated that GRM was a significantly better fitting model than GPCM (*p* < 0.001).

The DIF and item fit of the 12 AHS physical function at home items were evaluated using the GRM. All showed no significant DIF across age groups, tenure type, or income groups. Two items (Item 1: has difficulty getting into or out of bathtub, and Item 6: has difficulty reaching kitchen cabinets) showed significant DIF for sex (*p* < 0.001). In order to prevent biased estimation in functional difficulty at home, we separated these two items (Item 1 and Item 6) into distinct items by sex (i.e., Item 1 for males and Item 1 for females, Item 6 for males and Item 6 for females), resulting in a total of 14 AHS physical function at home items.

The GRM model fit to our new 14 AHS physical function at home items was reevaluated. The GRM on the items showed great model fit indices: RMSEA (0.029), CFI (0.996), and TLI (0.995), with all items greatly loading on the functional difficulty at home construct (λ > 0.79). Subsequent item-fit analyses indicated that Item 5 (has difficulty using fingers to grasp small objects), Item 8 (has difficulty reaching overhead), and Item 10 (has difficulty stooping or kneeling or bending) were statistically significant misfit items (*p* < 0.001). However, the magnitude of item misfit for all three items was negligible (RMSEA S-X2 < 0.013). Thus, we kept all 14 items for estimating older adults’ functional difficulty at home.

### 3.4. Measurement Validation

A total of 8542 older adults in the testing dataset were used for model validation. All 14 AHS items showed the expected hierarchy of item difficulty. As we hypothesized, the most challenging item to perform was a gross motor-associated task (Item 10, stooping or kneeling or bending), with the least challenging item being a fine motor-related task (Item 9, using sink) (for item difficulty, see Table 3). Item 11 (using stove), Item 2 (using kitchen counters), and Item 3 (using faucets) were in an error range of Item 9 (−2.50~−2.30). In addition, a large gap between the most (Item 10) and the second most (Item 1) challenging items was identified.

Factor scores of functional difficulty at home were estimated for each older adult using our final model. Factor scores ranged from −0.17 to 2.82, with 3457 older adults having the minimum factor scores. All 28 demographic and housing-related variables showed statistically significant differences for older adult functional difficulty at home (*p* < 0.001) (The means of each group’s functional difficulty and the F-statistics results are shown in the Appendix A). Controlling for age, female older adults had statistically significantly greater functional difficulty at home than male older adults (*p* < 0.0001).

## 4. Discussion

This study presents the process of using the 12 AHS physical function at home items to estimate older adults’ scores of functional difficulty at home. The results of this study demonstrated an adequate validity of the estimated factor scores of older adults’ functional difficulty, indicating that those factor scores are appropriate to use in other studies. The two subsequent studies will use machine learning to develop predictive models for identifying older adults at risk of experiencing functional difficulty at home. The factor scores estimated in the present study will serve as an output variable with a number of older adults’ demographic and housing-related conditions as input variables for the predictive models.

Approximately 40% of the older adults in our sample had the minimum factor score, indicating that they had no difficulty in performing all AHS physical function at home items. Including more challenging items would improve the measurements of physical difficulty at home for older adults and across a broader range of functional difficulty at home. However, since we aimed to estimate older adults’ functional difficulty at home in order to prevent possible home accidents, we expect that having a floor effect would not create complications for our study and reasoned that the estimated factors are appropriate for use in subsequent studies.

Item difficulties ranged from −2.40 to −0.96, illustrating that older adults with an average level of functional difficulty will have a greater probability of accomplishing all AHS physical function at home items. Except for Item 10 (stooping or kneeling or bending), all the items were in the error ranges with their adjacent items and ranged from −1.53 to −2.4. This indicates that a fewer number of items can be selected to develop a short form without sacrificing the measurement accuracy. We recommend further studies to investigate creating a short form of the AHS physical function at home items.

In the process of refining the measurement model, we separated Item 1 (Has difficulty getting into or out of bathtub) and Item 6 (Has difficulty reaching kitchen cabinets) for each sex due to their DIF impact. The subsequent analysis with the separated items showed that the two female items (Items 1 and 6 for females) were more challenging to succeed in than the two male items (Items 1 and 6 for males), even after controlling for sex differences in functional difficulty at home. This result indicates that, compared to male older adults, female older adults with the same level of functional difficulty at home experience relatively greater challenges in getting in and out of the bathtub (Item 1) and reaching kitchen cabinets (Item 6).

Women had a higher average factor score of functional difficulty at home than men (*p* < 0.001). Liang and his colleagues (2008) reported that women have greater and faster functional decline after age 50 than men [38]. A study published in the *Journal of NeuroEngineering and Rehabilitation* compared older adults’ (75–98 years) sex differences in functional mobility [39]. The study found that female older adults performed statistically significantly worse than male older adults in the following areas: completing coordinated stability, near tandem balance, walking speed, sit to stand, stair ascent and descent, and alternate step tasks (*p* < 0.05) [39]. Moreover, Kim and Ahrentzen (2016) showed that women were more likely to experience severe falling injuries than men [40]. Our study results align with these findings; however, why those two specific items (Items 1 and 6) are more difficult for female older adults than male older adults after controlling for their sex difference in functional difficulty levels is unknown. Future studies are recommended to examine the DIF in these two items between males and females.

Older adults showed statistically significant differences in their functional difficulty at home across all 28 demographic and housing-related variables, satisfying our hypotheses. This result confirms that different conditions of those variables are statistically associated with older adults’ functional difficulty at home. For example, as expected, older adults with excellent general health had an −0.02 average factor score of functional difficulty at home, whereas the score was 0.87 for older adults with poor general health. This finding verifies that our factor scores are valid to use in our subsequent studies.

### Limitation

This study has a few limitations. Our study used listwise deletion for the CFA, assuming that the missing is completely at random, which might have caused systematic bias in the CFA results. However, less than 3% of our sample was dropped from the analysis; thus, we suspect that the leverage of the possible bias was limited. Moreover, we used the 2011 AHS because health-related functional difficulty items were not available in more recent AHS surveys. The older adults’ home environment could have been altered over the last ten years, which could result in different outcomes. Nonetheless, in the 2019 AHS report, over 42% of residents had resided in their units for more than 10 years [41]. We expect that the impact was minimal.

## 5. Conclusions

Although survey items are often extracted and used to estimate an outcome of interest in general, they were not developed as a measurement tool. Therefore, carefully examining a factor structure and selecting a proper measurement model are essential steps for the accurate estimation of outcomes when using survey items. In this study, we demonstrated the process of using AHS physical function at home items to estimate functional difficulty at home for older adults, and we validated the resulting factor scores for use in subsequent studies. We encourage future studies to consider our method of using survey items to estimate an outcome of interest.

## Figures and Tables

**Table 1:** Response Frequency of American Housing Survey Physical Function at Home Items.

Items	Description	Category	Count (%)
Item 1	Has difficulty getting into or out of bathtub	No	25,716 (90.3%)
		Yes	2187 (7.7%)
		Missing	571 (2.0%)
Item 2	Has difficulty using kitchen counters	No	27,613 (97.0%)
		Yes	313 (1.1%)
		Missing	548 (1.9%)
Item 3	Has difficulty using faucets	No	27,658 (97.1%)
		Yes	270 (0.9%)
		Missing	546 (1.9%)
Item 4	Has difficulty getting to bathroom	No	27,436 (96.4%)
		Yes	491 (1.7%)
		Missing	547 (1.9%)
Item 5	Has difficulty using fingers to grasp small objects	No	26,201 (92.0%)
		Yes	1692 (5.9%)
		Missing	581 (2.0%)
Item 6	Has difficulty reaching kitchen cabinets	No	25,546 (89.7%)
		Yes	2378 (8.4%)
		Missing	550 (1.9%)
Item 7	Has difficulty opening kitchen cabinets	No	27,161 (95.4%)
		Yes	766 (2.7%)
		Missing	547 (1.9%)
Item 8	Has difficulty reaching over head	No	25,963 (91.2%)
		Yes	1931 (6.8%)
		Missing	580 (2.0%)
Item 9	Has difficulty using sink	No	27,674 (97.2%)
		Yes	256 (0.9%)
		Missing	544 (1.9%)
Item 10	Has difficulty stooping or kneeling or bending	No	22,156 (77.8%)
		Yes	5742 (20.2%)
		Missing	576 (2.0%)
Item 11	Has difficulty using stove	No	27,531 (96.7%)
		Yes	390 (1.4%)
		Missing	553 (1.9%)
Item 12	Has difficulty getting into or out of walk-in shower	No	26,946 (94.6%)
		Yes	959 (3.4%)
		Missing	569 (2.0%)

**Table 2:** Sample Characteristics.

Variable	Description	Category	Count (%)
Age Group	Age of person (years)	≥65 and <75	15,203 (53.4%)
		≥75 and <84	9286 (32.6%)
		≥85	3985 (14.0%)
Sex	What is the sex of this person: Male or Female?	Male	11,158 (39.2%)
		Female	17,316 (60.8%)
Tenure	The family income recode is the sum of the wage and salary income of the householder and all related individuals age 14+ and all other reported income	Owned	21,022 (73.8%)
		Rented	6996 (24.6%)
		Occupied without payment of rent	456 (1.6%)
Family Income Group	The family income recode is the sum of the wage and salary income of the householder and all related individuals age 14+ and all other reported income	≤25,000	12,911 (45.3%)
		>25,000	15,563 (54.7%)
Self-Care	Does anyone in this household have serious difficulty dressing or bathing?	Yes	1272 (4.5%)
		No	26,639 (93.6%)
		Missing	563 (2.0%)
Walking	Does anyone in this household have serious difficulty walking or climbing stairs?	Yes	6018 (21.1%)
		No	21,899 (76.9%)
		Missing	557 (2.0%)
General Health	Would you say that the head of household’s health in general is excellent, very good, fair, or poor?	Excellent	5712 (20.1%)
		Very Good	13,109 (46.0%)
		Fair	6987 (24.5%)
		Poor	2070 (7.3%)
		Missing	596 (2.1%)

**Table 3:** Item Difficulties of 14 American Housing Survey Physical Function at Home Items.

Item Description	Item Difficulty	S.E. *
Item 10_Stooping	−0.96	0.02
Item 1_F_Bathtub	−1.53	0.03
Item 6_F_Reaching Cabinet	−1.55	0.04
Item 1_M_Bathtub	−1.73	0.05
Item 8_Overhead	−1.77	0.03
Item 6_M_Reaching Cabinet	−1.8	0.05
Item 12_Shower	−1.97	0.04
Item 5_Small Object	−2.05	0.05
Item 7_Opening Cabinets	−2.1	0.04
Item 4_Bathroom	−2.16	0.04
Item 11_Stove	−2.31	0.05
Item 2_Counters	−2.39	0.05
Item 3_Faucets	−2.39	0.05
Item 9_Sink	−2.4	0.05

* Standard Error of Item Difficulty Estimates.

## Data Availability

Not applicable.

## Appendix A

**Table A1:** Estimated Home Functional Difficulty Factor Scores across Demographic and Housing-Related Variable Categories.

Variable	Description	Category	Mean	F-Statistic	*p*-Value
Self-Care	Does anyone in this household have serious difficulty dressing or bathing?	Yes	1.66	2508.2	<0.001
		No	0.13		
Walking	Does anyone in this household have serious difficulty walking or climbing stairs?	Yes	0.94	4831.02	<0.001
		No	0.01		
Age Group	Age of a person (years)	≥65 and <75	0.08	246.59	<0.001
		≥75 <84	0.24		
		≥85	0.53		
Sex	What is the sex of this person: Male or Female?	Male	0.10	128.48	<0.001
		Female	0.26		
Tenure	The family income recode is the sum of the wage and salary income of the householder and all related individuals age 14+ and all other reported income.	Owned	0.12	151.33	<0.001
		Rented	0.40		
		Occupied without payment of rent	0.29		
Income Group	The family income recode is the sum of the wage and salary income of the householder and all related individuals age 14+ and all other reported income.	≤25,000	0.31	249.91	<0.001
		>25,000	0.09		
General Health	Would you say that the head of household’s health in general is excellent, very good, fair, or poor?	Excellent	−0.02	434.36	<0.001
		Very Good	0.09		
		Fair	0.38		
		Poor	0.87		
Ramp	Does your home currently have any of the following features? Does anyone in the household currently use this feature on a regular basis because of a physical limitation? 1: Home has feature, and is currently used by a household member with a physical limitation, 2: Home has feature, but is not currently used by a household member with a physical limitation, 3: Home does not have feature	1	1.42	56.27	<0.001
		2	0.18		
		3	0.19		
Elevator		1	1.36	31.12	<0.001
		2	−0.02		
		3	0.19		
Bedroom on entry level		1	0.99	385.15	<0.001
		2	0.14		
		3	0.07		
Full bathroom on entry level		1	0.98	406.66	<0.001
		2	0.12		
		3	0.13		
Handrails or grab bars in unit		1	0.80	171.36	<0.001
		2	0.04		
		3	0.20		
Handrails or grab bars in bathroom		1	0.79	845.12	<0.001
		2	0.09		
		3	0.07		
Handrails or grab bars in other areas		1	0.76	83.68	<0.001
		2	0.11		
		3	0.18		
Built-in shower seats		1	0.89	264.14	<0.001
		2	0.10		
		3	0.16		
Raised toilets		1	0.84	351.17	<0.001
		2	0.12		
		3	0.15		
Door handles instead of knobs		1	1.03	198.87	<0.001
		2	0.13		
		3	0.18		
Sink handles/levers		1	1.04	302.65	<0.001
		2	0.14		
		3	0.17		
Extra-wide doors/hallways		1	1.10	238.51	<0.001
		2	0.19		
		3	0.17		
Kitchen trays/lazy susans		1	0.86	97.29	<0.001
		2	0.07		
		3	0.22		
No steps between rooms		1	0.98	664.06	<0.001
		2	0.12		
		3	0.12		
Wheelchair-accessible electrical outlets		1	1.14	326.95	<0.001
		2	0.15		
		3	0.18		
Wheelchair-accessible electrical switches		1	1.19	357.95	<0.001
		2	0.16		
		3	0.15		
Wheelchair-accessible climate controls		1	1.15	264.44	<0.001
		2	0.15		
		3	0.18		
Wheelchair-accessible kitchen cabinets		1	1.09	108.42	<0.001
		2	0.13		
		3	0.19		
Wheelchair-accessible countertops		1	1.21	302.49	<0.001
		2	0.16		
		3	0.16		
Wheelchair-accessible kitchen		1	1.15	196.12	<0.001
		2	0.16		
		3	0.18		
Wheelchair-accessible bathroom		1	1.12	309.18	<0.001
		2	0.13		
		3	0.19		

## References

[1] Fielo S.B., Warren S.A. (2001). Home Adaptation: Helping Older People Age in Place. Geriatr. Nurs..

[2] Housen P., Shannon G.R., Simon B., Edelen M.O., Cadogan M.P., Sohn L., Jones M., Buchanan J.L., Saliba D. (2008). What the Resident Meant to Say: Use of Cognitive Interviewing Techniques to Develop Questionnaires for Nursing Home Residents. Gerontologist.

[3] Keglovits M., Stark S. (2020). Home Modifications to Improve Function and Safety in the United States. J. Aging Environ..

[4] Sattin R.W. (1992). Falls among Older Persons: A Public Health Perspective. Annu. Rev. Public Health.

[5] Sattin R.W., Rodriguez J.G., DeVito C.A., Wingo P.A., Study to Assess Falls Among the Elderly (SAFE) Group (1998). Home Environmental Hazards and the Risk of Fall Injury Events among Community-Dwelling Older Persons. J. Am. Geriatr. Soc..

[6] Tinetti M.E., Williams C.S. (1997). Falls, Injuries Due to Falls, and the Risk of Admission to a Nursing Home. N. Engl. J. Med..

[7] Tinetti M.E., Speechley M., Ginter S.F. (1988). Risk Factors for Falls among Elderly Persons Living in the Community. N. Engl. J. Med..

[8] Romero S., Lee M.J., Simic I., Levy C., Sanford J. (2018). Development and Validation of a Remote Home Safety Protocol. Disabil. Rehabil. Assist. Technol..

[9] Clemson L., Fitzgerald M.H., Heard R. (1999). Content Validity of an Assessment Tool to Identify Home Fall Hazards: The Westmead Home Safety Assessment. Br. J. Occup. Ther..

[10] Tomita M.R., Saharan S., Rajendran S., Nochajski S.M., Schweitzer J.A. (2014). Psychometrics of the Home Safety Self-Assessment Tool (HSSAT) to Prevent Falls in Community-Dwelling Older Adults. Am. J. Occup. Ther..

[11] Fjell A., Cronfalk B.S., Carstens N., Rongve A., Kvinge L.M.R., Seiger Å., Skaug K., Boström A.-M. (2018). Risk Assessment during Preventive Home Visits among Older People. J. Multidiscip. Healthc..

[12] Iwarsson S. (2005). A Long-Term Perspective on Person–Environment Fit and ADL Dependence among Older Swedish Adults. Gerontologist.

[13] Hwang E., Cummings L., Sixsmith A., Sixsmith J. (2011). Impacts of Home Modifications on Aging-in-Place. J. Hous. Elder..

[14] Gillespie L.D., Robertson M.C., Gillespie W.J. (2009). Interventions for Preventing Falls in Older People Living in the Community. Cochrane Database Syst Rev.

[15] Nikolaus T., Bach M. (2003). Preventing Falls in Community-Dwelling Frail Older People Using a Home Intervention Team (HIT): Results from the Randomized Falls-HIT Trial. J. Am. Geriatr. Soc..

[16] Sanford J.A., Hoenig H., Griffiths P.C., Butterfield T., Richardson P., Hargraves K. (2007). A Comparison of Televideo and Traditional In-Home Rehabilitation in Mobility Impaired Older Adults. Phys. Occup. Ther. Geriatr..

[17] Wilson R.D., Lewis S.A., Murray P.K. (2009). Trends in the Rehabilitation Therapist Workforce in Underserved Areas: 1980–2000. J. Rural Health.

[18] Healthcare Access in Rural Communities Overview-Rural Health Information Hub. https://www.ruralhealthinfo.org/topics/healthcare-access#barriers.

[19] Northridge M.E., Nevitt M.C., Kelsey J.L., Link B. (1995). Home Hazards and Falls in the Elderly: The Role of Health and Functional Status. Am. J. Public Health.

[20] Tommasini C., Talamini R., Bidoli E., Sicolo N., Palese A. (2008). Risk Factors of Falls in Elderly Population in Acute Care Hospitals and Nursing Homes in North Italy: A Retrospective Study. J. Nurs. Care Qual..

[21] Chien W.-C., Lai C.-H., Chung C.-H., Lin C.-H. (2013). A Retrospective Population-Based Data Analyses of Unintentional Fall Mortality and Hospitalisation in Taiwan during 2005–2007. Int. J. Inj. Control Saf. Promot..

[22] American Housing Survey (AHS). https://www.census.gov/programs-surveys/ahs.html.

[23] Raykov T., Marcoulides G.A. (2011). Introduction to Psychometric Theory.

[24] Rosseel Y. (2011). lavaan: An R Package for Structural Equation Modeling and More Version 0.4-9 (BETA).

[25] Dominic C. Summarytools: Tools to Quickly and Neatly Summarize Data. https://cran.r-project.org/web/packages/summarytools/summarytools.pdf.

[26] Chalmers R.P. (2012). Mirt: A Multidimensional Item Response Theory Package for the R Environment. J. Stat. Softw..

[27] Chalmers R.P. (2016). Generating Adaptive and Non-Adaptive Test Interfaces for Multidimensional Item Response Theory Applications. J. Stat. Softw..

[28] Wickham H., François R., Henry L., Müller K. Dplyr: A Grammar of Data Manipulation. https://cran.r-project.org/web/packages/dplyr/dplyr.pdf.

[29] Wickham H. Tidyr: Tidy Messy Data. https://cran.r-project.org/web/packages/tidyr/tidyr.pdf.

[30] Ewing M. Mgsub: Safe, Multiple, Simultaneous String Substitution. https://cran.r-project.org/web/packages/mgsub/mgsub.pdf.

[31] R Core Team (2020). R: A Language and Environment for Statistical Computing. R Foundation for Statistical Computing.

[32] Weston S., Microsoft Foreach: Provides Foreach Looping Construct. https://cran.r-project.org/web/packages/foreach/foreach.pdf.

[33] Weston S., Microsoft DoParallel: Foreach Parallel Adaptor for the “Parallel” Package. https://cran.r-project.org/web/packages/doParallel/doParallel.pdf.

[34] Hooper D., Coughlan J., Mullen M.R. (2008). Structural Equation Modelling: Guidelines for Determining Model Fit. Electron. J. Bus. Res. Methods.

[35] Reeve B.B., Hays R.D., Bjorner J.B., Cook K.F., Crane P.K., Teresi J.A., Thissen D., Revicki D.A., Weiss D.J., Hambleton R.K. (2007). Psychometric Evaluation and Calibration of Health-Related Quality of Life Item Banks: Plans for the Patient-Reported Outcomes Measurement Information System (PROMIS). Med. Care.

[36] Linacre J.M. DIF-DPF-Bias-Interaction Concepts 2016. https://www.winsteps.com/winman/difconcepts.htm.

[37] Schuller W. (2020). Musculoskeletal Medicine in The Netherlands: Characteristics of Patients and Physicians, and Validity of Outcome Measurement Instruments.

[38] Liang J., Bennett J.M., Shaw B.A., Quiñones A.R., Ye W., Xu X., Ofstedal M.B. (2008). Gender Differences in Functional Status in Middle and Older Age: Are There Any Age Variations?. J. Gerontol. Ser. B Psychol. Sci. Soc. Sci..

[39] Butler A.A., Menant J.C., Tiedemann A.C., Lord S.R. (2009). Age and Gender Differences in Seven Tests of Functional Mobility. J. NeuroEngineering Rehabil..

[40] Kim D., Ahrentzen S. (2017). Environmental and Behavioral Circumstances and Consequences of Falls in a Senior Living Development. J. Hous. Elder..

[41] American Housing Survey Table Creator. https://www.census.gov/programs-surveys/ahs/data/interactive/ahstablecreator.html?s_areas=00000&s_year=2019&s_tablename=TABLE1&s_bygroup1=1&s_bygroup2=1&s_filtergroup1=1&s_filtergroup2=1.

